# Pneumatic elastostatics of multi-functional inflatable lattices: realization of extreme specific stiffness with active modulation and deployability

**DOI:** 10.1098/rsos.231272

**Published:** 2024-02-14

**Authors:** P. Sinha, T. Mukhopadhyay

**Affiliations:** ^1^ Department of Aerospace Engineering, Indian Institute of Technology Kanpur, Kanpur, India; ^2^ School of Engineering, University of Southampton, Southampton, UK

**Keywords:** inflatable lattices, active lattice materials, in-plane elastic properties, deployable lattices, on-demand property modulation

## Abstract

As a consequence of intense investigation on possible topologies of periodic lattices, the limit of specific elastic moduli that can be achieved solely through unit cell-level geometries in artificially engineered lattice-based materials has reached a point of saturation. There exists a robust rationale to involve more elementary-level mechanics for pushing such boundaries further to develop extreme lightweight multi-functional materials with adequate stiffness. We propose a novel class of inflatable lattice materials where the global-level stiffness can be derived based on a fundamentally different mechanics compared with conventional lattices having beam-like solid members, leading to extreme specific stiffness due to the presence of air in most of the lattice volume. Furthermore, such inflatable lattices would add multi-functionality in terms of on-demand performances such as compact storing, portability and deployment along with active stiffness modulation as a function of air pressure. We have developed an efficient unit cell-based analytical approach therein to characterize the effective elastic properties including the effect of non-rigid joints. The proposed inflatable lattices would open new frontiers in engineered materials and structures that will find critical applications in a range of technologically demanding industries such as aircraft structures, defence, soft robotics, space technologies, biomedical and various other mechanical systems.

## 1. Introduction

Artificially engineered materials can achieve a wide range of tailor-made multi-functional abilities which may not always be available in naturally occurring materials [1–5]. Their micro-scale design can present unprecedented and unconventional, yet useful, properties like ultra-lightweight characteristics [6–9], shape programming [6,10], crushing resistance and high specific energy absorption [11–14], auxetic properties [15–19], negative elastic moduli [20–22], meta-fluid characteristics [23,24], negative mass density [25,26], tunable wave propagation characteristics and vibration control [27–29], programmable constitutive laws [30–32], active mechanical property modulation [33–37] and many other multi-physical properties [38–42]. The use of such metamaterials in structural systems can result in the most optimal use of materials along with fulfilling multiple other structural demands simultaneously. Compared with conventional fibre-reinforced composite materials, in these architected materials there is a greater scope of modulating and improving the properties due to more elementary design possibility in an expanded design space [43–46]. With the progress in manufacturing technology, such complex microstructures are becoming easier to produce at the industrial scale. In this article, we intend to focus on developing a new class of inflatable [47–51] lattices with extreme specific stiffness and other on-demand multi-functional abilities [52–59]. We review some of the recent developments reported in the literature concerning lattice materials in the following paragraph, followed by a brief background on the rationale behind the current investigation.

High specific stiffness is essential for lightweight structures that find their usage in various mechanical and aerospace components such as sandwich structures [60–62]. Honeycomb-like hexagonal structural forms are adopted in a range of high-end engineering applications, besides these structures being common in various natural systems like wood and bone microstructures, nanostructures of two-dimensional materials like graphene and hexagonal boron nitride (hBN), etc. [63,64]. These hexagonal lattices have received immense attention in the last decade as one of the most extensively investigated topologies for auxetic and non-auxetic metamaterial development [65–67]. The unit cell-based method (i.e. representative volume element) is normally adopted to explore the effective elastic constants of periodic lattice materials, wherein a representative volume is analysed with appropriate periodic boundary conditions [68–71]. Recently, multi-material lattices [72] and voltage-dependent active periodic lattices [73] have been proposed in the literature. The impact of irregularity on the mechanical behaviour of lattice materials has been investigated [64,65,74–76]. The effect of pre-stress and residual stresses in the effective elastic moduli of honeycomb lattices have been characterized [77]. Recent studies involving nonlinear mechanics show that the introduction of anti-curvature in the microstructural design of metamaterials can significantly enhance the effective elastic moduli [78,79]. Besides investigating a range of periodic geometries as repeating units [80], researchers have also proposed hierarchical topologies in the design of metamaterials [81].

Investigating the effective elastic properties of engineered lattice-based materials is essential to utilize these microstructures for structural applications in various mechanical and aerospace systems. Besides enhancing the elastic moduli, many of such structural systems demand lightweight characteristics simultaneously. Owing to intense investigation on the possible topologies of these periodic lattices, the limit of specific elastic moduli that can be achieved solely through unit cell-level geometries has reached a point of saturation. Thus, there exists a robust rationale for involving more elementary-level mechanics for pushing the boundaries further to develop extreme lightweight multi-functional materials with adequate stiffness. Here we aim to propose a novel class of inflatable lattice-based materials where the global-level stiffness can be derived depending on a fundamentally different mechanics compared with conventional lattices with beam-like solid connecting members, leading to extreme specific stiffness due to the presence of air in most of the lattice volume. Further, such inflatable lattices would add multi-functionality in terms of on-demand performances such as compact storing, portability and deployment along with active stiffness modulation as a function of air pressure.

Normally, modulating the stiffness of the lattice structures after manufacturing is a challenging process. In conventional lattices with solid beams, once the lattice is manufactured, the effective elastic properties are fixed based on the microstructural geometries. However, there are advanced applications where the stiffness of the lattice needs to be actively modulated. For example, while one might need a stiffer lattice for reducing structural deflections, less stiffness is suitable for enhancing energy absorption and actuation capabilities. Inflatable lattices would introduce the possibility of on-demand stiffness modulation as a function of the internal air pressure. Further, it would be possible to store the lattice in compacted volume for transportation (using no air pressure) and later deployment during operation. For space (lightweight panels can be designed that can unfurl as and when required) and biomedical (such as stent development) applications, these capabilities can be quite attractive.

In this paper, we would propose inflatable lattices where the effective elastic properties can be modulated in an expanded microstructural design space including air pressure, fabric properties of the constituting inflatable beam elements and unit cell geometries. Generalized analytical formulae will be developed for characterizing the effective elastic properties (including the effect of non-rigid joints, which is conventionally neglected in the analysis of solid honeycomb lattices), followed by in-depth numerical analyses.

**Figure 1.**
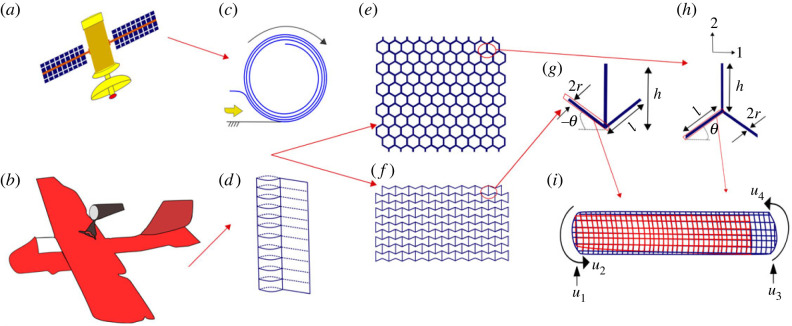
**Bottom-up analysis of inflatable lattices based on unit cell based approach**. (*a*) Prospective application of inflatable lattices in satellite structures such as deployable solar array paddles and structural booms. (*b*) Prospective application of inflatable lattices in inflatoplane, an all-fabric inflatable aircraft. In such a structure, when the fabric is exposed to air, it absorbs and repels water as it stiffens, giving the aircraft its shape and rigidity. (*c*,*d*) Coiling and compact storage of the inflatable lattices. The lattice can be deployed from the coiled stage when air pressure is increased. (*e*) A standard regular hexagonal honeycomb lattice geometry with positive Poisson’s ratio. (*f*) A typical auxetic lattice geometry with negative Poisson’s ratio. (*g*) Representative auxetic unit cell of the auxetic lattice structure. (*h*) Representative honeycomb unit cell of the regular hexagonal honeycomb structure. (*i*) Degrees of freedom of a typical beam-like inflatable member that forms the representative unit cell. Note that a periodic network of such beams essentially forms the entire lattice. Here we adopt a bottom-up approach for analysing the effective elastic moduli of lattice materials. We have considered the bending deformation along with the impact of internal gas pressure in the local deformation analysis of beams. Thereafter, a unit cell, made up of beam-like elements, is analysed for obtaining the formulae of the five in-plane elastic properties. At this stage, the impact of the geometry of the microstructure of the lattice is incorporated into the analysis. The effective elastic properties derived considering a single unit cell could be used subsequently in the design of engineering structures like space systems and aircraft. Note that the stiffness of the inflatable lattice can be actively modulated (on-demand) as a function of the air pressure.

The Timoshenko beam model for inflatable beams will be coupled with unit cell-based approach including the effect of joint rotation for developing the analytical formulation. While we aim to focus primarily on hexagonal lattices in this study (refer to figure 1*e*,*f*), the proposed concept of inflatable lattices will be applicable to other periodic topologies as well. Figure 1*a*–*i* shows the bottom-up framework, which shows the analysis from elementary inflatable beams to the unit cell levels and further to the lattice (auxetic and non-auxetic configurations) and structures level. Figure 1*c*,*d* presents a schematic representation of the capability of compact storage and deployment.

The present article is structured in the following sections hereafter, §2: theoretical derivation of the formulae of the five in-plane elastic constants of inflatable lattices considering internal pressure (including the effect of non-rigid joints), §3: numerical investigation considering different lattice geometries and internal pressure, §4: impact of the current study and concluding remarks.

## 2. Theoretical derivation of the effective elastic properties of inflatable lattices

We derive the effective elastic properties of inflatable hexagonal lattices in this section incorporating the effect of internal pressure. For the detailed derivation, a bottom-up approach is followed considering general hexagonal unit cells as shown in figure 1. First, we derive the formulation for transverse bending deflection of a cylindrical inflatable beam [82–86] (refer to figure 1*i*). Thereafter, we analyse a unit cell (imposing periodic boundary conditions) made up of inflatable beam elements for obtaining the closed-form analytical formulations of the five in-plane elastic properties of a lattice. The effect of the geometry of the lattice microstructure is considered at this point (refer to figure 1*g*,*h*). The homogenized effective elastic moduli, thus derived considering a unit cell, depict the global-level properties of the lattice material (refer to figure 1*e*) that could subsequently be used in designing larger structures (refer to figure 1*a*,*b*).

### 2.1. Mechanics of inflatable beams

**Figure 2.**
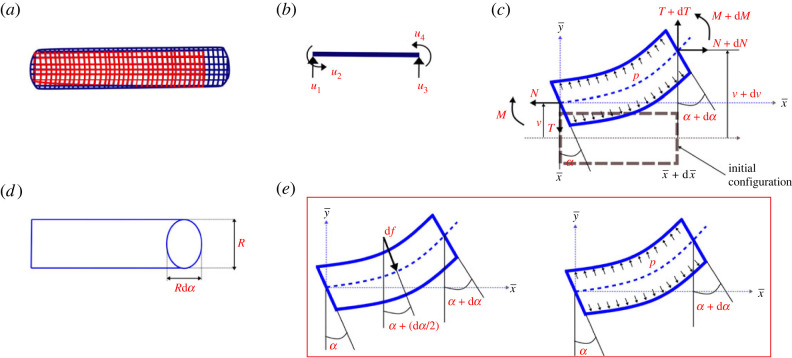
**Deformation mechanics of inflatable beams**. (*a*) Typical shape of an inflatable beam in undeformed condition. Note that the red colour denotes a beam with very low air pressure, while the blue colour denotes the pressurized beam configurations (without any other external transverse mechanical load). (*b*) Degrees of freedom of a beam-like single cell element. (*c*) Free body diagram of an inflated tube element. (*d*) Top view after deflection. (*e*) Force density due to the inflation pressure applied on the lateral walls, showing equivalent force from internal pressure.

In this section, we derive the deformation expressions for an inflated beam with appropriate boundary conditions. We use Timoshenko’s beam theory as we intend to account for the transverse shear effect for a more accurate analysis. The beam structure is made of an isotropic fabric membrane. For the beam model that we are considering, the internal pressure cannot be zero for deflection analysis. However, when the internal pressure is zero, it can be stored in a compact volume without the necessity of any deformation analysis (note the discussion on deployability in the preceding section). The pressure should be high enough to prevent wrinkling of the structure. This pressure is assumed to be uniform throughout the tube and the tube is of a circular cross-section in the current analysis. The beam cross-section, after deformation, is assumed to have the same shape as before deformation, and it remains perpendicular to the beam axis. Let *l* be the inflatable beam’s length and *r* be the radius of the beam cross-section. The beam is fixed at one end and we apply a concentrated force *F* at the other end (refer to figure 2*a*, showing the inflatable beam for visualization, wherein appropriate loading and boundary conditions are applied subsequently). If a beam has circular cross-section of radius *r* and membrane thickness *t* (assuming *t* ≪ *r*), we have *S* = 2*πrt* and *I* = *πr*^3^*t* as the cross-sectional area and the moment of inertia, respectively. We denote the lattice depth in direction orthogonal to plane of the panel by 2*r*. In the derivation, the intrinsic Young’s modulus of the beam elements of a unit cell is denoted by *E*, and their lengths are considered as *l* and *h* as per their alignment (refer to figure 1*g*,*h*). Since there exists closed end of the beams along the edge of the lattice, the pressure force *P*, due to the internal pressure *p* is given as

2.1 $$P=p\pi r^{2}.$$

This internal pressure is assumed to be a part of the structure. In our proposed model, the local beam axis is denoted by $\bar{x}$ and the transverse direction is denoted by $\bar{y}$. We will consider a small element at distance $\bar{x}$ from the fixed end (refer to figure 2). Here, $N(\bar{x})$, $T(\bar{x})$, $M(\bar{x})$, $v(\bar{x})$, $\alpha (\bar{x})$ and *p* represent the resultant axial stress, resultant shear stress, bending moment, deflection of the section centre, angle of rotation and internal pressure, respectively. Note that the radius of the small element *r* is same as that of the beam cross-section.

In the small element, we replace the pressure effects with forces applied normally to the internal solid cross-sectional area of the tube, making the fabrics pre-stressed. The projection from $\bar{y}$-direction is an ellipse after the deflection (refer to figure 2*c*,*d*). Therefore, the additional force in $\bar{y}$-direction is given as

2.2 $$df\;(\bar{y})=p\;dS=p\pi r^{2}\;d\alpha =P\;d\alpha .$$

The equivalent force density due to pressure on a side wall in the $\bar{y}$-direction is given by *P* = *pπr*^2^.

The equilibrium equations of the inflatable beam model based on the above discussion (refer to figure 2*c*) are given as

2.3a $$(N+dN)-N+df\;(\bar{y})\alpha =0,$$

2.3b $$(T+dT)-T-df\;(\bar{y})=0$$

2.3c $$\mathrm{and}\;\;\;\;\;\;(M+dM)-M-(N+dN)dv+(T+dT)\;d\bar{x}=0.$$

Equations (2.3*a*) and (2.3*b*), when integrated, give

2.4a $$N={\bar{C}}_{1}$$

and

2.4b $$T=P\alpha +{\bar{C}}_{2},$$

where ${\bar{C}}_{1}$ and ${\bar{C}}_{2}$ are the constants of integration. Let *E* be the elastic modulus and *G* be the shear modulus of the beam material. The value of shear factor, *K*, is taken to be 0.5 using Cowper’s theory for circular beam section [87]. In the present work, the inflatable beam material is assumed to have isotropic constitutive relation. As we are considering the shear effect of the inflatable beam, we use the Timoshenko beam theory for the small element. Since, after deformation, the beam’s section and the longitudinal axis do not remain perpendicular to each other, Euler–Bernoulli theory cannot be used for an accurate analysis here. Thus, we get

2.5a $$T=KGS\left(\frac{dv}{d\bar{x}}-\alpha \right)$$

and

2.5b $$M=EI\frac{d\alpha }{d\bar{x}}$$

Combining equations (2.4*b*) and (2.5*a*), we get

2.6 $$\frac{dv}{d\bar{x}}=\left(\frac{P}{KGS}+1\right)\alpha +\frac{{\bar{C}}_{2}}{KGS}.$$

The equations (2.4*a*), (2.4*b*) and (2.6), are now put in (2.3*c*). This leads to the final differential equation

2.7 $$EI\frac{d^{2}\alpha }{d{\bar{x}}^{2}}-\left(N+\frac{N-KGS}{KGS}P\right)\alpha -\frac{N-KGS}{KGS}{\bar{C}}_{2}=0.$$

To obtain ${\bar{C}}_{1}$ and ${\bar{C}}_{2}$, we impose the following boundary conditions first at $\bar{x}=0\;:\;$

2.8a α(0)=0,v(0)=0

2.8b T(0)=F,N(0)=P

2.8c $$\;M(0)=Fl\;\text{and}\;\frac{d\alpha }{d\bar{x}}(0)=\frac{M(0)}{EI}.$$

These constraints, when put in equations (2.4*a*,*b*), give

2.9a $${\bar{C}}_{1}=P$$

and

2.9b $${\bar{C}}_{2}=F$$

Equation (2.4*a*) shows that normal resultant stress (*N*) is constant, i.e. $N(\bar{x})=P$.

When the boundary conditions are put in the differential equation, the equation gets simplified, which gives the rotation of the section.

2.10 $$\frac{d^{2}\alpha }{d{\bar{x}}^{2}}-\frac{P^{2}}{KEIGS}\alpha =\frac{F}{EI}\left(\frac{P-KGS}{KGS}\right).$$

The solution of the differential equation is given as

2.11 $$\alpha (\bar{x})=A_{1}\;e^{g\bar{x}}+A_{2}e^{-g\bar{x}}+\delta_{1}.$$

Here the constants *A*_1_ and *A*_2_ are obtained using additional boundary conditions as given in the following paragraph, while *g* and *δ*_1_ are given as

2.12a $$g=\frac{P}{\sqrt{KEIGS}}$$

and

2.12b $$\delta_{1}=\frac{KGS-P}{P^{2}}F$$

The deflection of the inflated tube is then obtained as

2.13 $$v(\bar{x})=\frac{1}{g}\left(\frac{KGS+P}{KGS}\right)(A_{1}\;e^{g\bar{x}}-A_{2}\;e^{-g\bar{x}})+\frac{KGS}{KGS-P}\delta_{1}\bar{x}+\delta_{2},$$

where *δ*_2_ is a constant.

**Figure 3.**
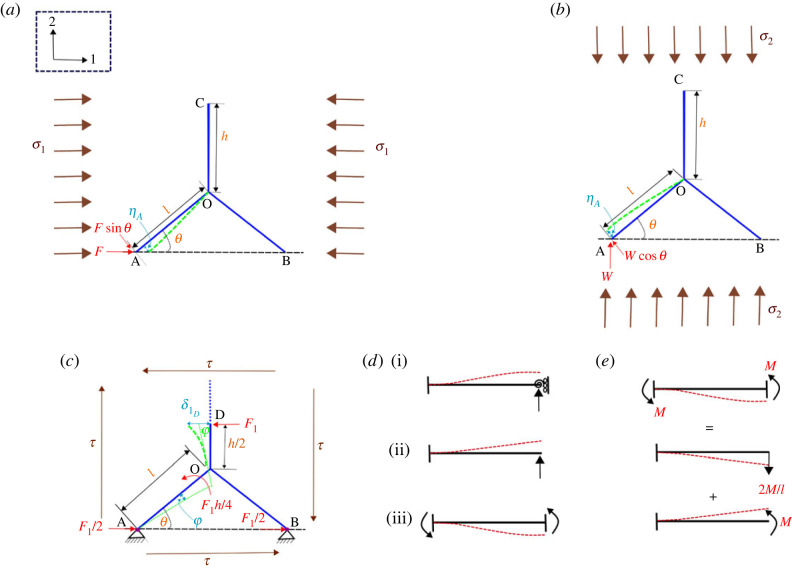
**Unit cell-level mechanics under normal and shear stresses**. (*a*) Analysis of a unit cell when the stress field *σ*_1_ is applied in the longitudinal direction. The closed-form expressions of the longitudinal Young’s modulus *E*_1_ and Poisson’s ratio *ν*_12_ are derived using this configuration. (*b*) Analysis of a unit cell when the stress field *σ*_2_ applied in the transverse direction. The closed-form expressions of the transverse Young’s modulus *E*_2_ and Poisson’s ratio *ν*_21_ are derived using this configuration. (*c*) Analysis of a unit cell when the stress (shear) field *τ* is applied. This configuration is used for obtaining the closed-form expression of shear modulus *G*_12_. (*d*) Beams with three different boundary conditions, which are required for lattice-level analysis. (i) Beam with one end fixed and no rotation allowed at the other. (ii) Beam with one end fixed and other free. (iii) Beam with both ends fixed and support sinks at one end. (*e*) Beam with support sinking that can be split into two cantilever beams with different loading conditions using superposition principle, one cantilever beam with load at the free end and the other one with a moment at its free end. The bold lines in the figures denote the initial shape of beam elements and the dotted lines represent the deformed shape due to the application of different loading conditions.

We can have additional boundary conditions of the inflated beams besides having one end clamped as considered in the preceding paragraphs. To maintain the periodic lattice-level boundary condition of the unit cells, the constituting beams need to have the remaining side, where the transverse load *F* is applied, rotationally restrained (refer to figure 3*d*(i)).

2.14 v(0)=0,α(0)=0andα(l)=0,

*v*(0) = 0 gives

2.15 $$\delta_{2}=-\frac{1}{g}\left(\frac{KGS+P}{KGS}\right)(A_{1}-A_{2}),$$

*α*(0) = 0 implies

2.16 $$A_{1}+A_{2}+\delta_{1}=0$$

and *α*(*l*) = 0 leads to

2.17 $$A_{1}\;e^{gl}+A_{2}\;e^{-gl}+\delta_{1}=0.$$

The above equations give *A*_1_ and *A*_2_ as

2.18a $$A_{1}=\frac{\delta_{1}(e^{-gl}-1)}{2\sinh gl}$$

and

2.18b $$A_{2}=\frac{\delta_{1}(1-e^{gl})}{2\sinh gl}.$$

The deflection at the end with the load *F* is then given as

2.19 $$v(l)=2F\frac{\left(K^{2}G^{2}S^{2}-P^{2}\right)}{gKGSP^{2}}\frac{\left(1-\cosh gl\right)}{\sinh gl}+\frac{KGS}{P^{2}}Fl.$$

We observe that pressure appears in the deflection values of the inflatable beam. Also, we can conclude from the above equation that in the case of inflatable beams, since they are made of the membrane, the pressure should not be zero, else the beam (or the connecting members of a lattice under consideration here) will collapse. The proposed derivation is valid as long as there is no wrinkle in the membrane, because the principal stress at any arbitrary point of the beam membrane needs to be non-negative. The analytical formulation developed in this section for inflatable beams will be used in the following sections for deriving the expressions for lattice-level elastic moduli following the unit cell-based approach.

### 2.2. Formulations for in-plane elastic moduli of inflatable hexagonal lattices

The material properties of a lattice material are defined at two different length scales. At the elementary level, the intrinsic properties are defined for the beams making up the lattice material. In this article, these elementary properties are the mechanical properties of the fabrics of the inflatable tube. In a much larger scale, the effective elastic moduli (*E*_1_, *E*_2_, *ν*_12_, *ν*_21_ and *G*_12_) of the lattice can further be defined to represent an homogenized effect. The homogenized effective moduli, thus obtained here, are functions of not only the geometry of the lattice but also of the intrinsic material properties and the air pressure. Therefore, even if the intrinsic material does not change, still at the global lattice level we can obtain different effective moduli as per the geometry of the lattice and air pressure. It can be highlighted that the current work proposes the design parameter in terms of internal air pressure in addition to the conventional lattice topology of the constituting beam-like members, leading to the capability of active stiffness modulation.

#### 2.2.1. Longitudinal Young’s modulus *E*_1_

To obtain the expression of effective longitudinal Young’s modulus, the unit cell is subjected to a remote uniform stress *σ*_1_ in direction-1. The stress produces force *F* on the unit cell at points A and B (refer to figure 3*a*). The relation between the force *F* on point A and the applied stress *σ*_1_ is given as

2.20 $$F=2\sigma_{1}r(h+l\sin\theta ).$$

The transverse deformation of the slant member AO is denoted by *η*_*A*_. This deformation, which occurs due to the force component Fsin⁡θ, can be expressed (using equation (2.19)) as

2.21 $$\begin{array}{rl} \eta_{A} & =v(l){|}_{F=F\sin\theta }\\ & =\left(2F\sin\theta \frac{\left(K^{2}G^{2}S^{2}-P^{2}\right)}{gKGSP^{2}}\frac{\left(1-\cosh gl\right)}{\sinh gl}+\frac{KGS}{P^{2}}Fl\sin\theta \right)\\ & =2\sigma_{1}r(h+l\sin\theta )\left(\frac{2(K^{2}G^{2}S^{2}-P^{2})}{gKGSP^{2}}\frac{\left(1-\cosh gl\right)}{\sinh gl}+\frac{KGS}{P^{2}}l\right)\sin\theta . \end{array}$$

Total deflection in direction-1 is thus given as

2.22 $$\begin{array}{rl} \delta_{1} & =\eta_{A}\sin\theta \\ & =\left(2F\frac{\left(K^{2}G^{2}S^{2}-P^{2}\right)}{gKGSP^{2}}\frac{\left(1-\cosh gl\right)}{\sinh gl}+\frac{KGS}{P^{2}}Fl\right)\sin^{2}\theta \\ & =2\sigma_{1}r(h+l\sin\theta )\left(\frac{2(K^{2}G^{2}S^{2}-P^{2})}{gKGSP^{2}}\frac{\left(1-\cosh gl\right)}{\sinh gl}+\frac{KGS}{P^{2}}l\right)\sin^{2}\theta . \end{array}$$

Strain along the direction of *σ*_1_ is given as

2.23 $$\begin{array}{rl} \varepsilon_{1} & =\frac{\delta_{1}}{l\cos\theta }\\ & =\frac{2\sigma_{1}r(h+l\sin\theta )\left(\frac{2(K^{2}G^{2}S^{2}-P^{2})}{gKGSP^{2}}\frac{\left(1-\cosh gl\right)}{\sinh gl}+\frac{KGS}{P^{2}}l\right)\sin^{2}\theta }{l\cos\theta }\\ & =2\sigma_{1}r\left(\frac{h}{l}+\sin\theta \right)\left(\frac{2(K^{2}G^{2}S^{2}-P^{2})}{gKGSP^{2}}\frac{\left(1-\cosh gl\right)}{\sinh gl}+\frac{KGS}{P^{2}}l\right)\sin\theta \tan\theta \end{array}$$

Young’s modulus (longitudinal direction) along the direction of the applied stress *σ*_1_ can now be expressed as

2.24 $$\begin{array}{rl} E_{1} & =\frac{\sigma_{1}}{\varepsilon_{1}}\\ & =\frac{\;p\pi \cos\theta }{2(\frac{h}{l}+\sin\theta )\sin^{2}\theta \left(\begin{array}{cc} \sqrt{\frac{2E}{KG}}{\left(4K^{2}(\frac{G}{p}\right)}^{2}{\left(\frac{t}{r}\right)}^{2}-1)\frac{\left(1-\cosh\left(\frac{\;p}{\sqrt{2KEG}}(\frac{l}{t})\right)\right)}{\sinh\left(\frac{\;p}{\sqrt{2KEG}}(\frac{l}{t})\right)} & \\ +\;2K(\frac{G}{p}){\left(\frac{t}{r}\right)}^{2}(\frac{l}{t}) \end{array}\right)} \end{array}$$

#### 2.2.2. Poisson’s ratio *ν*_12_

To obtain Poisson’s ratio *ν*_12_, we require an additional strain in the lateral direction (i.e. direction-2), arising as a result of *σ*_1_ that is applied along the longitudinal direction (refer to figure 3*a*). From equation (2.21) for the expression of the deformation, the total deflection in direction-2 (*δ*_2_) is obtained as

2.25 $$\begin{array}{rl} -\delta_{2} & =\eta_{A}\cos\theta \\ & =\left(2F\frac{\left(K^{2}G^{2}S^{2}-P^{2}\right)}{gKGSP^{2}}\frac{\left(1-\cosh gl\right)}{\sinh gl}+\frac{KGS}{P^{2}}Fl\right)\sin\theta \cos\theta \\ & =2\sigma_{1}r(h+l\sin\theta )\left(\frac{2(K^{2}G^{2}S^{2}-P^{2})}{gKGSP^{2}}\frac{\left(1-\cosh gl\right)}{\sinh gl}+\frac{KGS}{P^{2}}l\right)\sin\theta \cos\theta . \end{array}$$

The negative sign of *δ*_2_ is because this deflection is in opposite direction to the considered positive convention (expansion in this case). Total strain in the lateral direction (i.e. direction-2) is given as

2.26 $$\begin{array}{rl} -\varepsilon_{2} & =\frac{\delta_{2}}{h+l\sin\theta }\\ & =\frac{2\sigma_{1}r(h+l\sin\theta )\left(\frac{2(K^{2}G^{2}S^{2}-P^{2})}{gKGSP^{2}}\frac{\left(1-\cosh gl\right)}{\sinh gl}+\frac{KGS}{P^{2}}l\right)\sin\theta \cos\theta }{h+l\sin\theta }\\ & =2\sigma_{1}r\left(\frac{2(K^{2}G^{2}S^{2}-P^{2})}{gKGSP^{2}}\frac{\left(1-\cosh gl\right)}{\sinh gl}+\frac{KGS}{P^{2}}l\right)\sin\theta \cos\theta . \end{array}$$

Using equations (2.23) and (2.26), Poisson’s ratio *ν*_12_ is given as

2.27 $$\nu_{12}=-\frac{\varepsilon_{2}}{\varepsilon_{1}}=\frac{\cos^{2}\theta }{(\frac{h}{l}+\sin\theta )\sin\theta }.$$

#### 2.2.3. Transverse Young’s modulus *E*_2_

For obtaining the transverse Young’s modulus *E*_2_, a uniform remote stress *σ*_2_ is applied to the unit cell in direction-2. The resulting vertical force *W* acting on point A of a unit cell due to the applied stress is expressed as (refer to figure 3*b*)

2.28 $$W=2\sigma_{2}rl\cos\theta .$$

The lateral deformation of the slant member AO is denoted by *η*_*A*_. This deformation, which occurs due to the force component Wcos⁡θ, can be expressed (using equation (2.19)) as

2.29 $$\begin{array}{rl} \eta_{A} & =v(l){|}_{F=W\cos\theta }\\ & =\left(2W\cos\theta \frac{\left(K^{2}G^{2}S^{2}-P^{2}\right)}{gKGSP^{2}}\frac{\left(1-\cosh gl\right)}{\sinh gl}+\frac{KGS}{P^{2}}Wl\cos\theta \right)\\ & =2\sigma_{2}rl\cos^{2}\theta \left(\frac{2(K^{2}G^{2}S^{2}-P^{2})}{gKGSP^{2}}\frac{\left(1-\cosh gl\right)}{\sinh gl}+\frac{KGS}{P^{2}}l\right). \end{array}$$

The total deflection in the direction-2, i.e. the deflection of the slant member AO, is thereby given as

2.30 $$\begin{array}{rl} \delta_{2} & =\eta_{A}\cos\theta \\ & =2\sigma_{2}rl\cos^{3}\theta \left(\frac{2(K^{2}G^{2}S^{2}-P^{2})}{gKGSP^{2}}\frac{\left(1-\cosh gl\right)}{\sinh gl}+\frac{KGS}{P^{2}}l\right). \end{array}$$

The strain along *σ*_2_ is given by

2.31 $$\begin{array}{rl} \varepsilon_{2} & =\frac{\delta_{2}}{\left(h+l\sin\theta \right)}\\ & =\frac{2\sigma_{2}rl\cos^{3}\theta (\frac{2(K^{2}G^{2}S^{2}-P^{2})}{gKGSP^{2}}\frac{\left(1-\cosh gl\right)}{\sinh gl}+\frac{KGS}{P^{2}}l)}{\left(h+l\sin\theta \right)}. \end{array}$$

The effective Young’s modulus in direction-2 is thus given as

2.32 $$\begin{array}{rl} E_{2} & =\frac{\sigma_{2}}{\varepsilon_{2}}\\ & =\frac{\;p\pi (\frac{h}{l}+\sin\theta )}{2\cos^{3}\theta \left(\sqrt{\frac{2E}{KG}}(4K^{2}{\left(\frac{G}{p}\right)}^{2}{\left(\frac{t}{r}\right)}^{2}-1)\frac{\left(1-\cosh\left(\frac{\;p}{\sqrt{2KEG}}(\frac{l}{t})\right)\right)}{\sinh\left(\frac{\;p}{\sqrt{2KEG}}(\frac{l}{t})\right)}+2K(\frac{G}{p}){\left(\frac{t}{r}\right)}^{2}(\frac{l}{t})\right)} \end{array}$$

#### 2.2.4. Poisson’s ratio *ν*_21_

For deriving the Poisson’s ratio *ν*_21_, we additionally require the strain in direction-1, arising due to the stress *σ*_2_ that is applied in the transverse direction. From the equation (2.29) for the expression of the deformation, the total deflection in direction-1 (*δ*_1_) is obtained as (refer to figure 3*b*)

2.33 $$\begin{array}{rl} \delta_{1} & =-\eta_{A}\sin\theta \\ & =-2\sigma_{2}rl\cos^{2}\theta \left(\frac{2(K^{2}G^{2}S^{2}-P^{2})}{gKGSP^{2}}\frac{\left(1-\cosh gl\right)}{\sinh gl}+\frac{KGS}{P^{2}}l\right)\sin\theta . \end{array}$$

Thus, the strain in the axial direction (i.e. direction-1) can be expressed as

2.34 $$\begin{array}{rl} \varepsilon_{1} & =\frac{\delta_{1}}{l\cos\theta }\\ & =-2\sigma_{2}r\cos\theta \left(\frac{2(K^{2}G^{2}S^{2}-P^{2})}{gKGSP^{2}}\frac{\left(1-\cosh gl\right)}{\sinh gl}+\frac{KGS}{P^{2}}l\right)\sin\theta . \end{array}$$

From equations (2.31) and (2.34), the closed-form expression of Poisson’s ratio *ν*_21_ is given as

2.35 $$\nu_{21}=-\frac{\varepsilon_{1}}{\varepsilon_{2}}=\frac{((h/l)+\sin\theta )\sin\theta }{\cos^{2}\theta }.$$

We can conclude from the expressions in (2.27) and (2.35), that Poisson’s ratio is independent of the internal gas pressure *p*. It depends only on the geometry of the unit cell. This observation is in accordance with our previous studies [73,88,89], where the effect of other mechanisms of deformation are considered besides axial deformation and static bending (like the effects of electrical field and ambient vibration).

#### 2.2.5. Shear modulus *G*_12_

The closed-form expression of the shear modulus *G*_12_ can be derived by considering the shear strain arising because of the bending deformation of vertical member along with the effect of the joint rotation (refer to figure 3*c*). The deflection of adjacent cells accounting for the bending deformations is considered in figure 3*c*, which shows that the midpoint of the vertical member will deform only along direction-1 due to shear (the bending moment at the midpoint becomes zero due to the boundary and loading conditions). The unit cell shown in figure 3*c* comprised the vertical member and the slant member of lengths *h*/2 and *l*, respectively. The relative movement between points O and A is negated because of symmetry.

The shear deformation owing to bending is denoted by *γ*. It takes into account the bending deflection of the member OD and the joint rotation of O due to the bending of the inclined members. For deriving the bending deformation (transverse to the longitudinal beam axis) of point D with respect to point O in direction-1, we consider an inflatable cantilever tube of length *h*/2 and calculate the deflection due to the concentrated force *F*_1_ acting at the free end. In §2.1, we have derived the deflection of a beam under the boundary condition as: one end fixed and the other end rotationally restrained. For deriving *G*_12_, in addition to this boundary condition, we also need beams with cantilever boundary condition as: one end fixed and the other end free. The beam boundary conditions in the cantilever case are given as

2.36 $$v(0)=0,\;\alpha (0)=0\;\text{and}\;M\left(\frac{h}{2}\right)=0,$$

*v*(0) = 0 gives

2.37 $$\delta_{2}=-\frac{1}{g}\left(\frac{KGS+P}{KGS}\right)(A_{1}-A_{2}),$$

*α*(0) = 0 implies

2.38 $$A_{1}+A_{2}+\delta_{1}=0,$$

$M(\frac{h}{2})=0$ gives

2.39 $$A_{1}e^{gh/2}=A_{2}\;e^{-gh/2},$$

*A*_1_ and *A*_2_ are then given as

2.40a $$A_{1}=-\frac{\delta_{1}(e^{-gh/2})}{2\cosh(gh/2)}$$

and

2.40b $$A_{2}=-\frac{\delta_{1}(e^{gh/2})}{2\cosh(gh/2)}.$$

The deflection at the end D with the load *F*_1_ is then given as

2.41 $$\eta_{d}=-F_{1}\frac{\left(K^{2}G^{2}S^{2}-P^{2}\right)}{gKGSP^{2}}\tanh\left(\frac{gh}{2}\right)+F_{1}\frac{KGS}{P^{2}}\frac{h}{2},$$

where *F*_1_ is given as

2.42 $$F_{1}=4\tau lr\cos\theta .$$

Moment that acts at point O (refer to figure 3*c*(a)) is given by

2.43 $$M=\frac{F_{1}}{2}\times \frac{h}{2}=\frac{F_{1}h}{4}.$$

Now we require the deflection at O due to moment *M* at O. We will apply the superposition principle for this under the assumption of small deformation. For this, we follow the same approach as in the calculation of deflection of an inflatable cantilever tube, with the only added condition of a moment *M* along with a concentrated load *F*, both at the free end. The boundary conditions for the analytical expression of deflection at free end of a cantilever beam are given as

2.44 v(0)=0andα(0)=0.

One addition to the above boundary conditions due to application of the moment *M* at free end is (we superimpose the conditions for a cantilever beam with a force and a moment at the free end)

2.45 $$M(l)=EI\frac{d\alpha (l)}{d\bar{x}},$$

which leads to

2.46 $$A_{1}e^{gl}-A_{2}\;e^{-gl}=\frac{M}{EIg}.$$

*A*_1_ and *A*_2_ are then given as

2.47a $$A_{1}=\frac{1}{2\cosh gl}\left(-\delta_{1}e^{-gl}+\frac{M}{EIg}\right)$$

and

2.47b $$A_{2}=\frac{1}{2\cosh gl}\left(-\delta_{1}e^{gl}-\frac{M}{EIg}\right).$$

The deflection value for the inflatable beam with concentrated load *F* and moment *M* at the free end is then given as

2.48 $$v(l)=F\left(\frac{KGS}{P^{2}}\left(l-\frac{\tanh gl}{g}\right)+\frac{\tanh gl}{KGSg}\right)+M\left(\left(\frac{KGS+P}{P^{2}}\right)\left(1-\frac{1}{\cosh gl}\right)\right).$$

In our case, the direction of rotation is opposite to the considered sign convention. Thus, to calculate the deflection due to rotation for this inflatable beam, we consider the force value as −2*M*/*l* and moment as *M* and put it in equation (2.48) (refer to figure 3*e*). Thus, deflection of end O (due to the application of moment *M* at the end O) with respect to the end A is

2.49 $$\begin{array}{rl} \delta_{r} & =-\frac{2M}{l}\left(\frac{KGS}{P^{2}}\left(l-\frac{\tanh gl}{g}\right)+\frac{\tanh gl}{KGSg}\right)+M\left(\left(\frac{KGS+P}{P^{2}}\right)\left(1-\frac{1}{\cosh gl}\right)\right)\\ & =M(-\frac{2}{l}\left(\frac{KGS}{P^{2}}\left(l-\frac{\tanh gl}{g}\right)+\frac{\tanh gl}{KGSg}\right)+\left(\left(\frac{KGS+P}{P^{2}}\right)\left(1-\frac{1}{\cosh gl}\right)\right)). \end{array}$$

Subsequently, rotation of point O is given by

2.50 $$\begin{array}{rl} \varphi & =\frac{\delta_{r}}{l}\\ & =\frac{M}{l}(-\frac{2}{l}\left(\frac{KGS}{P^{2}}\left(l-\frac{\tanh gl}{g}\right)+\frac{\tanh gl}{KGSg}\right)+\left(\left(\frac{KGS+P}{P^{2}}\right)\left(1-\frac{1}{\cosh gl}\right)\right))\\ & =\frac{F_{1}h}{4l}(-\frac{2}{l}\left(\frac{KGS}{P^{2}}\left(l-\frac{\tanh gl}{g}\right)+\frac{\tanh gl}{KGSg}\right)+\left(\left(\frac{KGS+P}{P^{2}}\right)\left(1-\frac{1}{\cosh gl}\right)\right)). \end{array}$$

The shear deformation along direction-1 due to shear stress *τ* is given by

2.51 $$\begin{array}{rl} \delta_{1_{D}} & =2\left(\varphi \frac{h}{2}+\eta_{D}\right)\\ & =\frac{F_{1}h^{2}}{4l}(-\frac{2}{l}\left(\frac{KGS}{P^{2}}\left(l-\frac{\tanh gl}{g}\right)+\frac{\tanh gl}{KGSg}\right)+\left(\left(\frac{KGS+P}{P^{2}}\right)\left(1-\frac{1}{\cosh gl}\right)\right))\\ & \;-F_{1}\frac{2(K^{2}G^{2}S^{2}-P^{2})}{gKGSP^{2}}\tanh\left(\frac{gh}{2}\right)+F_{1}\frac{KGSh}{P^{2}}. \end{array}$$

The total shear strain, *γ*, is given by

2.52 $$\begin{array}{rl} \gamma & =\frac{\delta_{1_{D}}}{h+l\sin\theta }\\ & =\frac{F_{1}}{h+l\sin\theta }\\ & \;\times (\left(\frac{h^{2}}{4l}\left(-\frac{2}{l}\left(\frac{KGS}{P^{2}}\left(l-\frac{\tanh gl}{g}\right)+\frac{\tanh gl}{KGSg}\right)+\left(\left(\frac{KGS+P}{P^{2}}\right)\left(1-\frac{1}{\cosh gl}\right)\right)\right)\right)\\ & \;-\frac{2(K^{2}G^{2}S^{2}-P^{2})}{gKGSP^{2}}\tanh\left(\frac{gh}{2}\right)+\frac{KGSh}{P^{2}}). \end{array}$$

The shear modulus in closed form is thus expressed as

2.53 $$\begin{array}{rl} G_{12} & =\frac{\tau }{\gamma }\\ & =\frac{\;p\pi (\frac{h}{l}+\sin\theta )}{\cos\theta \left(\begin{array}{cc} -4K(\frac{G}{p}){\left(\frac{h}{l}\right)}^{2}(\frac{t}{r})\left((\frac{l}{r})-\frac{\sqrt{2KEG}}{p}(\frac{t}{r})\tanh\left(\frac{\;p}{\sqrt{2KEG}}(\frac{l}{t})\right)\right) & \\ -\sqrt{\frac{2E}{KG}}{\left(\frac{h}{l}\right)}^{2}\tanh\left(\frac{\;p}{\sqrt{2KEG}}(\frac{l}{t})\right) & \\ +(\frac{h}{l})(\frac{h}{r})(2K(\frac{G}{p})(\frac{t}{r})+1)\left(1-\frac{1}{\cosh\left(\frac{\;p}{\sqrt{2KEG}}(\frac{l}{t})\right)}\right) & \\ -\sqrt{\frac{32E}{KG}}(4K^{2}{\left(\frac{G}{p}\right)}^{2}{\left(\frac{t}{r}\right)}^{2}-1)\tanh\left(\frac{\;p}{\sqrt{8KEG}}(\frac{h}{t})\right)+8K(\frac{G}{p})(\frac{t}{r})(\frac{h}{r}) \end{array}\right)}. \end{array}$$

### 2.3. Critical remarks

#### 2.3.1. Remark 1: no wrinkling condition

The presented derivation in this article is valid till the point when there are no wrinkles in the membrane since the principal stress at any arbitrary point of the membrane must not be negative. Note that such a condition can be easily maintained with sufficient air pressure and it does not create any hindrance in the proposed functionality of the inflatable lattices. We show the theoretical limits here to maintain the no-wrinkling condition. If the wall of the inflated beam does not wrinkle, then the minimum axial stress, *σ*_min_ in the inflated cylindrical beam is non-negative (*σ*_min_ ≥ 0) [90,91], where

2.54 $$\sigma_{\min}=\frac{\;pR}{2t}-\frac{M}{\pi R^{2}t}.$$

Here *p* is the internal pressure, *M* is moment due to the external load applied, *R* and *t* are radius and thickness of the beam, respectively. Thus, the applied far-field stresses and pressure should satisfy the following relations to avoid wrinkle:

2.55 $$\left(\frac{\sigma_{1}}{p}\right)\leq {\left(\frac{r}{l}\right)}^{2}\frac{\pi }{2(\frac{\frac{h}{l}}{+}\sin\theta )}$$

2.56 $$\left(\frac{\sigma_{2}}{p}\right)\leq {\left(\frac{r}{l}\right)}^{2}\frac{\pi }{2\cos\theta }$$

2.57 $$\mathrm{and}\;\;\;\;\;\;\;\;\;\left(\frac{\tau }{p}\right)\leq \left(\frac{r}{l}\right)\left(\frac{r}{h}\right)\frac{\pi }{4\cos\theta }.$$

Equation (2.57) considers the axial stress in both the members of the unit cell shown in figure 3*c*, i.e. the slant beam member of length *l* and the other vertical beam member of length *h*/2.

#### 2.3.2. Remark 2: reciprocal theorem

The closed-form formulations of the elastic constants derived above perfectly satisfy the reciprocal theorem [68]

2.58 $$\begin{array}{rl} E_{1}\nu_{21} & =E_{2}\nu_{12}\\ & =\frac{\;p\pi }{2\sin\theta \cos\theta \left(\begin{array}{cc} \sqrt{\frac{2E}{KG}}(4K^{2}{\left(\frac{G}{p}\right)}^{2}{\left(\frac{t}{r}\right)}^{2}-1)\frac{\left(1-\cosh\left((\;p/\sqrt{2KEG})(l/t)\right)\right)}{\sinh\left((\;p/\sqrt{2KEG})(l/t)\right)} & \\ +\;2K(\frac{G}{p}){\left(\frac{t}{r}\right)}^{2}(\frac{l}{t}) \end{array}\right)} \end{array}$$

The above condition, which is a classical knowledge in the field of solid mechanics, further adds confidence in the current derivation.

#### 2.3.3. Remark 3: in-plane elastic moduli for regular honeycombs

Honeycombs having *θ* = 30° and *h* = *l* have isotropic material properties. These regular honeycombs have wide industry usage. From the formulations derived in §2.2, the effective elastic properties of regular honeycombs (having *θ* = 30° and *h* = *l*) can be given as

2.59 $$\begin{array}{rl} \frac{E_{1}}{E} & =\frac{E_{2}}{E}\\ & =\frac{\left(\frac{\;p\pi }{E}\right)}{2\left(\sqrt{\frac{2E}{KG}}(4K^{2}{\left(\frac{G}{p}\right)}^{2}{\left(\frac{t}{r}\right)}^{2}-1)\frac{\left(1-\cosh\left((\;p/\sqrt{2KEG})(l/t)\right)\right)}{\sinh\left((\;p/\sqrt{2KEG})(l/t)\right)}+2K(\frac{G}{p}){\left(\frac{t}{r}\right)}^{2}(\frac{l}{t})\right)}. \end{array}$$

Since the values of effective Young’s moduli in longitudinal and transverse directions are the same, the regular hexagonal inflatable honeycombs are isotropic.

Similarly, the shear modulus of uniform inflatable honeycombs (*h* = *l* and *θ* = 30°) can be given as

2.60 $$\begin{array}{rl} \frac{G_{12}}{E} & =\frac{\left(\frac{\;p\pi }{E})(\frac{h}{l}+\sin\theta \right)}{\cos\theta \left(\begin{array}{c} -4K(\frac{G}{p}){\left(\frac{h}{l}\right)}^{2}(\frac{t}{r})\left((\frac{l}{r})-\frac{\sqrt{2KEG}}{p}(\frac{t}{r})\tanh\left(\frac{\;p}{\sqrt{2KEG}}(\frac{l}{t})\right)\right)\\ \;-\sqrt{\frac{2E}{KG}}{\left(\frac{h}{l}\right)}^{2}\tanh\left(\frac{\;p}{\sqrt{2KEG}}(\frac{l}{t})\right)\\ \;+(\frac{h}{l})(\frac{h}{r})(2K(\frac{G}{p})(\frac{t}{r})+1)\left(1-\frac{1}{\cosh\left(\frac{\;p}{\sqrt{2KEG}}(\frac{l}{t})\right)}\right)\\ \;-\sqrt{\frac{32E}{KG}}(4K^{2}{\left(\frac{G}{p}\right)}^{2}{\left(\frac{t}{r}\right)}^{2}-1)\tanh\left(\frac{\;p}{\sqrt{8KEG}}(\frac{h}{t})\right)+8K(\frac{G}{p})(\frac{t}{r})(\frac{h}{r}) \end{array}\right)} \end{array}$$

Note that value of Poisson’s ratios of regular uniform inflatable honeycombs (*θ* = 30° and *h* = *l*) become unity (i.e. *ν*_12_ = *ν*_21_ = 1).

#### 2.3.4. Remark 4: applicability to auxetic lattices

The effective elastic moduli of re-entrant auxetic lattices having negative Poisson’s ratios (refer to figure 1*f*) are quantified by changing the *θ* in the equations (2.24), (2.27), (2.32), (2.35) and (2.53) to −*θ*.

2.61 $$\begin{array}{rl} E_{1} & =\frac{\;p\pi \cos\theta }{2(\frac{h}{l}-\sin\theta )\sin^{2}\theta \left(\begin{array}{cc} \sqrt{\frac{2E}{KG}}(4K^{2}{\left(\frac{G}{p}\right)}^{2}{\left(\frac{t}{r}\right)}^{2}-1)\frac{\left(1-\cosh\left(\frac{\;p}{\sqrt{2KEG}}(\frac{l}{t})\right)\right)}{\sinh\left(\frac{\;p}{\sqrt{2KEG}}(\frac{l}{t})\right)}\\ & \;+2K(\frac{G}{p}){\left(\frac{t}{r}\right)}^{2}(\frac{l}{t}) \end{array}\right)} \end{array}$$

2.62 $$\nu_{12}=-\frac{\cos^{2}\theta }{(\frac{h}{l}-\sin\theta )\sin\theta }$$

2.63 $$E_{2}=\frac{\;p\pi ((h/l)-\sin\theta )}{2\cos^{3}\theta \left(\sqrt{\frac{2E}{KG}}(4K^{2}{\left(\frac{G}{p}\right)}^{2}{\left(\frac{t}{r}\right)}^{2}-1)\frac{\left(1-\cosh\left(\frac{\;p}{\sqrt{2KEG}}(\frac{l}{t})\right)\right)}{\sinh\left(\frac{\;p}{\sqrt{2KEG}}(\frac{l}{t})\right)}+2K(\frac{G}{p}){\left(\frac{t}{r}\right)}^{2}(\frac{l}{t})\right)}$$

2.64 $$\nu_{21}=-\frac{(\frac{h}{l}-\sin\theta )\sin\theta }{\cos^{2}\theta }$$

2.65 $$\begin{array}{r} \mathrm{and}\;G_{12}=\frac{\;p\pi \left(\frac{h}{l}-\sin\theta \right)}{\cos\theta \left(\begin{array}{c} 4K\left(\frac{G}{p}\right){\left(\frac{h}{l}\right)}^{2}\left(\frac{t}{r}\right)\left(\left(\frac{l}{r}\right)-\frac{\sqrt{2KEG}}{p}\left(\frac{t}{r}\right)\tanh\left(\frac{\;p}{\sqrt{2KEG}}\left(\frac{l}{t}\right)\right)\right)\\ \;+\sqrt{\frac{2E}{KG}}{\left(\frac{h}{l}\right)}^{2}\tanh\left(\frac{\;p}{\sqrt{2KEG}}\left(\frac{l}{t}\right)\right)\\ \;-\left(\frac{h}{l}\right)\left(\frac{h}{r}\right)\left(2K\left(\frac{G}{p}\right)\left(\frac{t}{r}\right)+1\right)\left(1-\frac{1}{\cosh\left(\frac{\;p}{\sqrt{2KEG}}\left(\frac{l}{t}\right)\right)}\right)\\ \;-\sqrt{\frac{32E}{KG}}\left(4K^{2}{\left(\frac{G}{p}\right)}^{2}{\left(\frac{t}{r}\right)}^{2}-1\right)\tanh\left(\frac{\;p}{\sqrt{8KEG}}\left(\frac{h}{t}\right)\right)+8K\left(\frac{G}{p}\right)\left(\frac{t}{r}\right)\left(\frac{h}{r}\right) \end{array}\right)} \end{array}$$

Note from the above equations that the numerical values of Poisson’s ratios (not dependent on air pressure) become negative for auxetic configurations.

#### 2.3.5. Remark 5: generalization of elastic moduli to consider hinging effect (non-rigid joints)

To accurately analyse the inflatable lattices, the influence of joint rotation (when the joints are not perfectly rigid) can further be incorporated along with transverse deflections by introducing hinging effect. If the mechanism of hinging [92] is also accounted for along with the bending mechanism, the elastic moduli are given as (refer to the electronic supplementary material for full derivation)

2.66 $$E_{1}=\frac{\cos\theta }{2(\frac{h}{l}+\sin\theta )\sin^{2}\theta \left(\begin{array}{cc} \frac{1}{p\pi }\sqrt{\frac{2E}{KG}}(4K^{2}{\left(\frac{G}{p}\right)}^{2}{\left(\frac{t}{r}\right)}^{2}-1)\frac{\left(1-\cosh\left(\frac{\;p}{\sqrt{2KEG}}(\frac{l}{t})\right)\right)}{\sinh\left(\frac{\;p}{\sqrt{2KEG}}(\frac{l}{t})\right)}\\ & +\frac{2K}{p\pi }(\frac{G}{p}){\left(\frac{t}{r}\right)}^{2}(\frac{l}{t})+\frac{rl^{2}}{2K_{\theta }} \end{array}\right)}$$

2.67 $$\nu_{12}=\frac{\cos^{2}\theta }{(\frac{h}{l}+\sin\theta )\sin\theta }$$

2.68 $$E_{2}=\frac{\left(\frac{h}{l}+\sin\theta \right)}{2\cos^{3}\theta \left(\begin{array}{c} \frac{1}{p\pi }\sqrt{\frac{2E}{KG}}(4K^{2}{\left(\frac{G}{p}\right)}^{2}{\left(\frac{t}{r}\right)}^{2}-1)\frac{\left(1-\cosh\left(\frac{\;p}{\sqrt{2KEG}}(\frac{l}{t})\right)\right)}{\sinh\left(\frac{\;p}{\sqrt{2KEG}}(\frac{l}{t})\right)}\\ +\frac{2K}{p\pi }(\frac{G}{p}){\left(\frac{t}{r}\right)}^{2}(\frac{l}{t})+\frac{rl^{2}}{2K_{\theta }} \end{array}\right)}$$

2.69 $$\nu_{21}=\frac{(\frac{h}{l}+\sin\theta )\sin\theta }{\cos^{2}\theta }$$

2.70 $$\mathrm{and}\;G_{12}=\frac{\left(\frac{h}{l}+\sin\theta \right)}{\cos\theta \left(\begin{array}{c} -\frac{4K}{p\pi }(\frac{G}{p}){\left(\frac{h}{l}\right)}^{2}(\frac{t}{r})\left((\frac{l}{r})-\frac{\sqrt{2KEG}}{p}(\frac{t}{r})\tanh\left(\frac{\;p}{\sqrt{2KEG}}(\frac{l}{t})\right)\right)\\ -\frac{1}{p\pi }\sqrt{\frac{2E}{KG}}{\left(\frac{h}{l}\right)}^{2}\tanh\left(\frac{\;p}{\sqrt{2KEG}}(\frac{l}{t})\right)\\ +(\frac{1}{p\pi })(\frac{h}{l})(\frac{h}{r})(2K(\frac{G}{p})(\frac{t}{r})+1)\left(1-\frac{1}{\cosh\left(\frac{\;p}{\sqrt{2KEG}}(\frac{l}{t})\right)}\right)\\ -(\frac{1}{p\pi })\sqrt{\frac{32E}{KG}}(4K^{2}{\left(\frac{G}{p}\right)}^{2}{\left(\frac{t}{r}\right)}^{2}-1)\tanh\left(\frac{\;p}{\sqrt{8KEG}}(\frac{h}{t})\right)+\frac{8K}{p\pi }(\frac{G}{p})(\frac{t}{r})(\frac{h}{r})\\ +\frac{3rh^{2}}{K_{\theta }} \end{array}\right)}$$

In the above expressions, *K*_*θ*_ denotes the rotational stiffness of joints against hinging. Interestingly, when *K*_*θ*_ → ∞, these expressions are found to be exactly similar to the expressions of elastic moduli derived in equations (2.24), (2.27), (2.32), (2.35) and (2.53).

#### 2.3.6. Remark 6: relative density of inflatable lattices

Relative density, *ρ*, of a honeycomb lattice is defined as the ratio of the density of the cellular material (i.e. honeycomb lattice) to the density of the solid intrinsic material of which the cell walls are composed. For our case, we derive the expression of the relative density of an inflatable honeycomb lattice by considering the unit cell shown in figure 1*h*. We write the expression of *ρ* in terms of volume of all the three members of the unit cell divided by the volume of the equivalent rectangular plate-like volume formed by the unit cell. Thus the expression of relative density for an inflatable honeycomb lattice is given by

2.71 $$\rho =\frac{(h+2l)2\pi rt}{(h+l\sin\theta )(2l\cos\theta )2r}=\frac{(\frac{h}{l}+2)\pi t}{2l(\frac{h}{l}+\sin\theta )\cos\theta }.$$

The geometrical parameters involved in the above expression are indicated in figure 1*g*,*h*.

### 2.4. Sensitivity analysis

The elastic moduli of inflatable honeycomb lattices are functions of the microstructural geometry, intrinsic material parameters and the air pressure, all of which have different relative sensitivity. Thus, it becomes necessary to characterize the relative sensitivities of different influencing parameters [93] on the five effective in-plane elastic properties under consideration. It could give a better understanding concerning the design of inflatable lattices. Here, the sensitivity analysis of elastic moduli is performed using the Monte Carlo simulation (MCS) approach, in which the closed-form expressions of the in-plane elastic moduli derived in equations (2.24), (2.27), (2.32), (2.35) and (2.53) are utilized.

In the current analysis, there are seven influencing input parameters, which are *E*, *t*/*l*, *h*/*l*, *θ*, *p*, *ν* and *r*/*l*. We use the MCS approach to calculate the mean and standard deviation corresponding to the individual variation of each of the input parameters, wherein different random combinations of the influencing factors are defined within a bound [94]. The standard deviation and mean for defining the bound of input parameter sets are related by the degree of randomness *s*_0_, i.e. *σ* = *μ* × *s*_0_. From the standard deviation and mean values for each set of MCS corresponding to individual variations of the input parameters, we obtain the sensitivity of the *i*th input parameter as

2.72 $$S_{i}=\frac{\left({\bar{c}}_{i}\right)}{\sum_{i=1}^{5}({\bar{c}}_{i})},$$

where

2.73 $$\sum_{i=1}^{5}S_{i}=1.$$

Here ${\bar{c}}_{i}$ quantifies the ratio of standard deviation and mean and is referred to as the coefficient of variation, which represents the case where only the *i*th input parameter is taken to be stochastic, while all other parameters are considered as deterministic. Note that we have adopted the MCS approach in our current analysis but the first-order perturbation technique (FOPT) and second-order perturbation technique (SOPT), can also be used to obtain the standard deviation and mean for small randomness. The MCS-based results are often considered as the baseline results as it does thousands of function evaluations numerically.

## 3. Results and discussion

In this section, we present numerical results to characterize the effect of air pressure and unit cell geometry in inflatable lattices based on the derived analytical formulae. However, prior to embarking on such numerical investigation, we first establish the validity of the expression of deflection of the inflatable beams with appropriate boundary conditions (refer to figure 3*d*), depending on which the elastic constants of a lattice material are further derived. To do so, all the three inflatable beams with internal pressure, as shown in figure 3*d*, are considered (refer to equations (2.19), (2.41) and (2.49)) and we have compared the results with the transverse deflections obtained using equation (3.1) [95,96].

3.1 $$\left[\begin{array}{c} K \end{array}\right]=\frac{\left(EI+\frac{PI}{S}\right)}{l^{3}(1+\phi )}\left[\begin{array}{cccc} 12 & 6l & -12 & 6l\\ 6l & l^{2}(4+\phi ) & -6l & l^{2}(2-\phi )\\ -12 & -6l_{0} & 12 & -6l_{0}\\ 6l & l^{2}(2-\phi ) & -6l & l^{2}(4+\phi ) \end{array}\right]$$

where

3.2 $$\phi =\frac{12(EI+\frac{PI}{S})}{(P+KGS)l^{2}},$$

**Figure 4.**
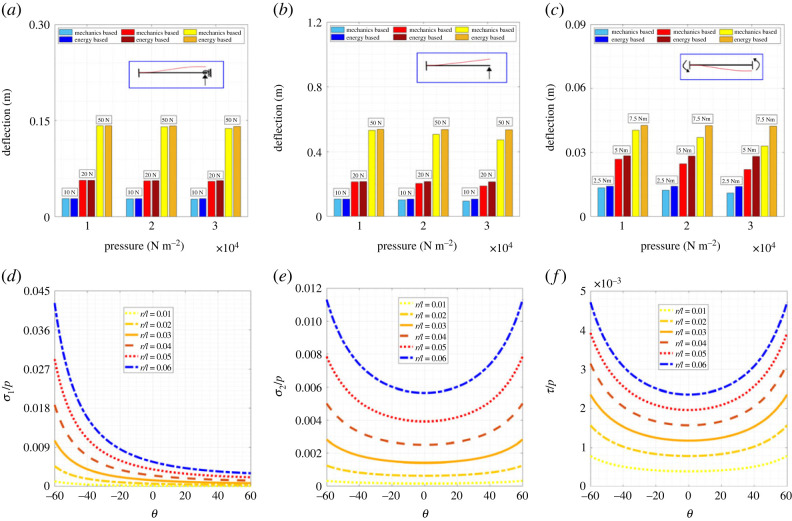
**Numerical validation for inflatable beams with different boundary conditions and permissible non-dimensional stress values in the beams under different lattice-level loading conditions**. (*a*) Validation of the beam with one end fixed and load at the other with no rotation allowed by comparing the deformation values calculated using vector mechanics-based approach and energy-based approach. (*b*) Validation of the beam with one end fixed and load at the other end which is free by comparing the deformation values calculated using vector mechanics-based approach and energy-based approach. (*c*) Validation of the support sinking beam with both ends fixed by comparing the deformation values calculated using vector mechanics-based approach and energy-based approach. The inflatable beam parameters considered are, length 1 m, radius 0.05 m and thickness 0.02 mm. (*d*) Variation of permissible non-dimensional stress along direction-1, *σ*_1_/*p* with *θ* at different values of *r*/*l*, so that no wrinkling occurs. (*e*) Variation of permissible non-dimensionalized stress along direction-2, *σ*_2_/*p* with *θ* at different values of *r*/*l*, so that no wrinkling occurs. (*f*) Variation of permissible non-dimensionalized shear stress, *τ*/*p* with *θ* at different values of *r*/*l*, so that no wrinkling occurs.

While our derivation is based on a vectorial mechanics approach, the stiffness matrix of equation (3.1) (taken from the above-mentioned literature) is obtained using energy-based principles. Thus comparison of the results for beam-level deflections, obtained using two different approaches, would serve as a validation of the current formulation. Figure 4*a*–*c* shows that the results obtained from the two approaches agree well for different internal pressure values and external lateral load and moment, establishing the validity of the closed-form expression of deflection deduced in equations (2.19), (2.41) and (2.49). Further, exact analytical validations and experimental validation can be obtained for expressions (2.41) and (2.49) with established literature [83]. Besides the beam-level validation, we have also validated the lattice-level framework for obtaining effective elastic moduli by considering the case of conventional solid beams, as available in literature [68]. For such a lattice configuration an exact analytical validation can be obtained for *K*_*θ*_ → ∞. After having the beam-level models validated and having confidence on the lattice-level characterization, in the subsequent paragraphs, we would characterize the effective elastic properties of inflatable lattice materials according to the analytical formulae derived in the present work.

As presented in equations (2.55)–(2.57), to avoid any wrinkle formation, a threshold value of internal pressure needs to be maintained depending on the nature and value of lattice-level remote stresses. Figure 4*d*–*f* presents the variation in stress applied for the evaluation of elastic constants of hexagonal inflatable lattices in different configurations obtained by varying the cell angle *θ*, analysed in conjunction with the change in *r*/*l* ratio. We have non-dimensionalized the results considering *σ*_1_/*p*, *σ*_2_/*p* and *τ*/*p* for the normal stresses along direction-1 and direction-2 and shear stress, respectively. These plots provide an idea about the allowable combination of permissible remote stress and air pressure for different lattice configurations.

**Figure 5.**
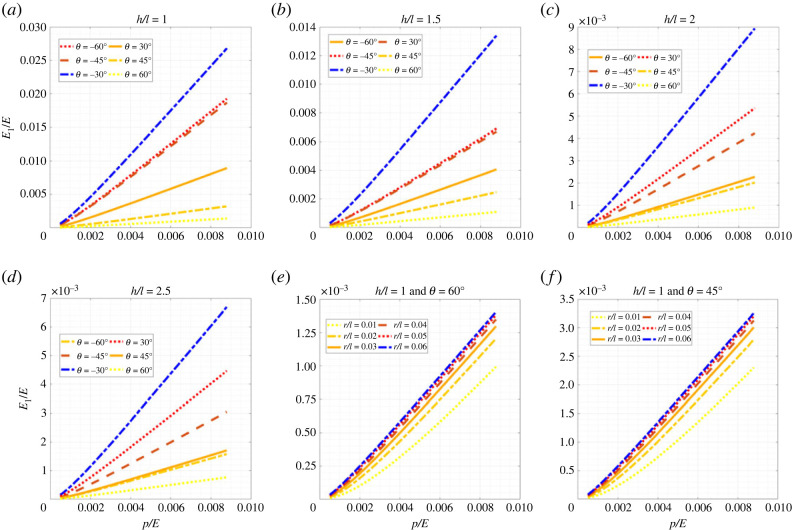
**Longitudinal effective Young’s modulus for hexagonal inflatable lattices under the influence of air pressure**. Variation of non-dimensionalized effective Young’s modulus, *E*_1_/*E*, with the non-dimensionalized internal pressure, *p*/*E*, (*a*) at different values of *θ* for *h*/*l* = 1, (*b*) at different values of *θ* for *h*/*l* = 1.5, (*c*) at different values of *θ* for *h*/*l* = 2, (*d*) at different values of *θ* for *h*/*l* = 2.5, (*e*) at different values of ratio of radius and length, *r*/*l* for *h*/*l* = 1 and *θ* = 60°, and (*f*) at different values of ratio of radius and length, *r*/*l* for *h*/*l* = 1 and *θ* = 45°.

**Figure 6.**
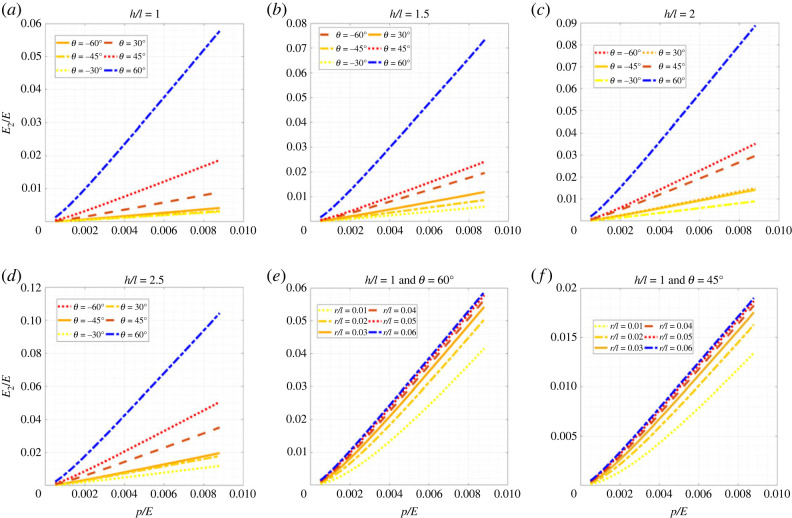
**Transverse effective Young’s modulus for hexagonal inflatable lattices under the influence of air pressure**. Variation of non-dimensionalized effective Young’s modulus, *E*_2_/*E*, with the non-dimensionalized internal pressure, *p*/*E*, (*a*) at different values of *θ* for *h*/*l* = 1, (*b*) at different values of *θ* for *h*/*l* = 1.5, (*c*) at different values of *θ* for *h*/*l* = 2, (*d*) at different values of *θ* for *h*/*l* = 2.5, (*e*) at different values of ratio of radius and length, *r*/*l* for *h*/*l* = 1 and *θ* = 60°, and (*f*) at different values of ratio of radius and length, *r*/*l* for *h*/*l* = 1 and *θ* = 45°.

**Figure 7.**
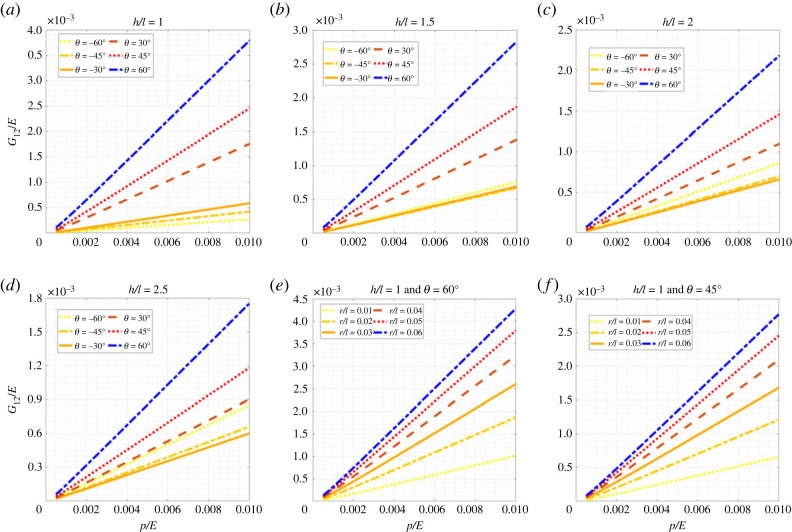
**In-plane effective shear modulus for hexagonal inflatable lattices under the influence of air pressure**. Variation of non-dimensionalized effective shear modulus, *G*_12_/*E*, with the non-dimensionalized internal pressure, *p*/*E*, (*a*) at different values of *θ* for *h*/*l* = 1, (*b*) at different values of *θ* for *h*/*l* = 1.5, (*c*) at different values of *θ* for *h*/*l* = 2, (*d*) at different values of *θ* for *h*/*l* = 2.5, (*e*) at different values of ratio of radius and length, *r*/*l* for *h*/*l* = 1 and *θ* = 60°, and (*f*) at different values of ratio of radius and length, *r*/*l* for *h*/*l* = 1 and *θ* = 45°.

Figures 5–7 show the change in elastic moduli of hexagonal lattices considering different unit cell geometries as a function of the internal pressure. Different configurations of both positive and negative inclination angle *θ* (non-auxetic and auxetic) are analysed considering the change in the ratio *h*/*l*. It can be noted in this context that the auxetic configurations are physically possible only when h/l≥2sin⁡θ. Thus, some of the results which are presented here for completeness, may not actually be possible to realize physically from a manufacturing point of view. Furthermore, the no-wrinkling condition, as discussed in remark 1, needs to be taken into consideration while deciding the range of applicability of the air pressure, which can be readily obtained using the derived closed-form analytical expressions. We have presented the results based on non-dimensional forms *E*_1_/*E*, *E*_2_/*E* and *G*_12_/*E* for the elastic moduli and *p*/*E* for the internal pressure. Here the intrinsic Young’s modulus of the material of which the beam is made is denoted by *E*. For each of the geometric configurations (i.e. both *θ* and *h*/*l*), with the increase in internal pressure, the elastic moduli increase and vice versa. This gives us the advantage of active modulation of elastic properties, i.e. we can modulate the values of elastic properties as a function of pressure. Normal lattices have constant properties after manufacturing; but here even after fabrication, we can modulate the effective elastic properties of the lattices, leading to the capability of on-demand stiffness control. From figures 5–7, we notice a general trend that the elastic modulus *E*_1_ reduces with the increase in cell angle and *h*/*l* ratio, while decreasing with *r*/*l* ratio. Similarly, *E*_2_ increases with the increase in cell angle, *h*/*l* and *r*/*l* ratios. The shear modulus *G*_12_ increases with the increase in cell angle and *r*/*l* ratio, while decreasing with *h*/*l* ratio. In this context, note that we have not presented numerical results concerning Poisson’s ratios here, since these are not a function of the air pressure, as evident from the closed-form formulae derived in the preceding section. However, we have shown the numerical results for Poisson’s ratio in the supplementary material.

**Figure 8.**
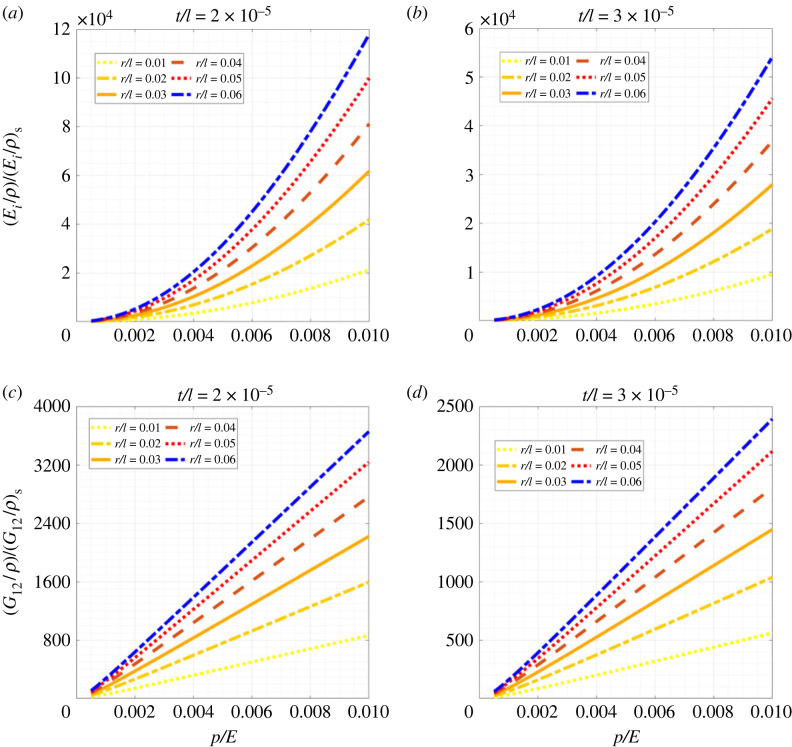
**Comparative assessment of specific elastic properties of inflatable lattices with respect to conventional solid lattices**. (*a*) Variation of ratio of Young’s modulus of an inflatable beam upon its relative density and Young’s modulus of a solid beam with circular cross-section upon its relative density, at different values of *r*/*l* for *t*/*l* = 2 × 10^−5^. (*b*) Variation of ratio of Young’s modulus of an inflatable beam upon its relative density and Young’s modulus of a solid beam with circular cross-section upon its relative density, at different values of *r*/*l* for *t*/*l* = 3 × 10^−5^. Here *i* = 1, 2, i.e. the variation is valid for both *E*_1_ and *E*_2_. (*c*) Variation of ratio of shear modulus of an inflatable beam upon its relative density and shear modulus of a solid beam with circular cross-section upon its relative density, at different values of *r*/*l* for *t*/*l* = 2 × 10^−5^. (*d*) Variation of ratio of shear modulus of an inflatable beam upon its relative density and shear modulus of a solid beam with circular cross-section upon its relative density, at different values of *r*/*l* for *t*/*l* = 3 × 10^−5^. Note that the subscript *s* is used to denote the solid beam.

The introduction of a fundamentally different mechanism for obtaining stiffness in lattice materials through air pressure leads to a dramatic increase in specific elastic properties (i.e. the ratio of elastic moduli and relative density, where the relative density is defined as the ratio of the volume of material in the hexagonal lattice and the total volume of an equivalent solid continuum throughout the domain [68]). To present the results in proper context, we make a comparison of the specific elastic moduli of inflated lattices and conventional solid lattices (note: the expressions for elastic moduli of lattices with solid circular beams are presented in the electronic supplementary material). Figure 8 shows the variation of the ratio of the specific elastic moduli for inflated lattices and conventional lattices as a function of the air pressure. Here we have non-dimensionalized the results considering (*E*_1_/*ρ*)/(*E*_1_/*ρ*)_*s*_, (*E*_2_/*ρ*)/(*E*_2_/*ρ*)_*s*_ and (*G*_12_/*ρ*)/(*G*_12_/*ρ*)_*s*_ for the elastic moduli and *p*/*E* for the internal pressure. The subscript *s* denotes the properties that correspond to that of a solid circular beam. As is evident from the plots, the values of (*E*_*i*_/*ρ*) (where *i* = 1, 2) and (*G*_12_/*ρ*) for the inflatable lattices are significantly higher (to the range of 10^3^) than that for the solid lattices. Such outcomes bring us to the realization of the importance of the current proposition of inflatable lattices in developing lightweight load-bearing structures.

**Figure 9.**
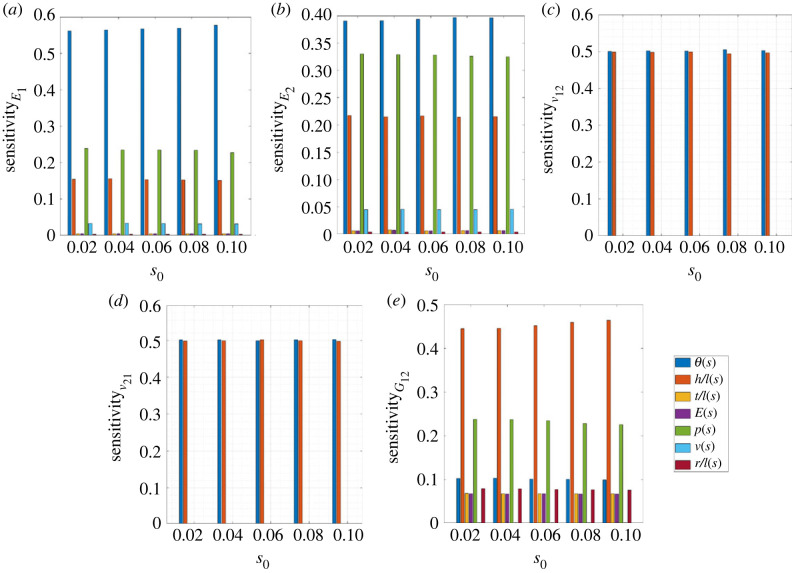
**Relative sensitivity of the air pressure, microstructural geometries and intrinsic material properties**. (*a*) Young’s modulus *E*_1_ in direction-1 (*b*) Young’s modulus *E*_2_ in direction-2 (*c*) Poisson’s ratio *ν*_12_ in 1-2 plane (*d*) Poisson’s ratio *ν*_21_ in 1-2 plane (*e*) In-plane effective shear modulus *G*_12_.

The elastic moduli of inflatable lattices depend on multiple parameters including the air pressure, unit cell geometry and intrinsic material properties. It is important to analyse the relative sensitivity of different input parameters for a better insight into the design process. As discussed in §2.4, we have numerically evaluated the relative sensitivity of different influencing factors on the elastic properties of inflated lattice materials using MCS. Figure 9 presents the relative sensitivity of different geometric parameters (*θ*, *h*/*l*, *t*/*l* and *r*/*l*), intrinsic material properties (*ν* and *E*) and the internal pressure (*p*) on the five elastic moduli. The results show that in general the geometric parameters and internal pressure are most sensitive for Young’s moduli and shear modulus. We further notice that the sensitivity of internal pressure and intrinsic material properties are zero for the two Poisson’s ratios since these are dependent only on the geometric parameters *θ* and *h*/*l*. Note here that the relative sensitivity for any particular elastic modulus is constant for varying degrees of randomness (*s*_0_), asserting the stability of the presented results.

## 4. Summary and perspective

A dramatic increase in the specific elastic moduli is reported in this article compared with conventional solid lattices based on a novel class of inflatable lattice metamaterials. We have proposed an impactful concept of developing lightweight honeycomb-based lattice metamaterials with such extreme mechanical properties through computational and analytical investigations, which are validated extensively at multiple levels using experimental and numerical results to garner adequate confidence and reproducibility. The derived explicit closed-form expressions for effective elastic moduli are presentable in the manuscript and suitable for writing straightforward and computationally efficient codes. Such expressions can provide clear insights readily into the effect of different influencing parameters (such as lattice geometry, intrinsic material properties, air pressure, etc.) on the effective elastic properties of inflatable lattices.

The Timoshenko beam theory is adopted, taking into account the bending and shear deformations along with the coupled effect of internal gas pressure for the local-level deformation mechanics of the beams. Subsequently, the effect of the geometry of lattice microstructure is integrated with the beam-level analysis following a unit cell-based approach to quantify the effective elastic moduli of the lattices. As an integral part of the study, the effect of non-rigid joints is further included in the analytical framework through a hinging mechanism at the joints for more practically relevant analyses.

The vector mechanics-based approach for the deformation of inflatable beams with different boundary conditions, which are subsequently utilized for obtaining the lattice-level effective properties, is validated extensively with the results of available in the literature. The lattice-level effective properties are validated with standard results from literature considering a special case of solid beams. The effective elastic properties at the lattice level conform to the reciprocal theorem, garnering further confidence to the developed formulation in addition to the two-stage validation at the beam and lattice levels.

The proposed inflatable lattices can achieve ∼10^3^ times higher specific elastic moduli compared with conventional solid lattices. The current investigation further reveals that the two in-plane Poisson’s ratios are not influenced by air pressure, while Young’s moduli and shear modulus can be controlled as a function of the air pressure for on-demand stiffness modulation including the extreme rise in their values. A Monte Carlo simulation-based relative sensitivity analysis confirms the effectiveness of air pressure in addition to the geometric parameters in modulating the elastic properties. Quantification of sensitivity as presented in this paper, even after having explicit closed-form equations, is useful for design and operational control purposes.

In general, lattice-based engineered materials are attracting significant attention in recent years due to their multi-functional abilities that can be tailored according to specific applications. Investigating the effective elastic properties of this new class of materials is essential to use these microstructures for structural applications in various mechanical and aerospace systems. Owing to intense investigation on the possible topologies of such periodic lattices, the limit of specific elastic moduli that can be achieved solely through unit cell-level geometries has reached a point of saturation. The current paper essentially addresses the strong rationale for pushing the boundaries further to develop extreme lightweight multi-functional materials with adequate stiffness by involving more elementary-level mechanics. In the proposed inflatable lattice materials, the global-level stiffness is derived based on a fundamentally different mechanics compared with conventional lattices with solid beam-like elements, leading to extreme specific stiffness due to the presence of air in most of the lattice volume.

Besides extreme specific stiffness, the inflatable lattices add multi-functionality in terms of on-demand performances such as compact storing, portability and deployment along with active stiffness modulation as a function of air pressure. Note that quantification of effective elastic properties at the global level can facilitate a range of analyses of the lattices such as deformations and stresses in different directions, low-frequency vibration and buckling analysis. The availability of a wide range of stiffness in the same structure (as a function of air pressure) can be ideal for reducing deflection under operating conditions to keep it under allowable limits, and increasing frequency to avoid resonance, while the same structure can be used for contradictory applications like high energy absorption by allowing large deformation. Such functionalities can be particularly attractive for technologically demanding applications like space structures, inflatable solar panels, deployable aircraft wings, soft robotics and biomedical applications. A range of new capabilities in the high-dimensional design space including active (air pressure) and passive (unit cell geometry with auxetic and non-auxetic configurations along with intrinsic material properties) parameters would essentially bring a step-change in the field of multi-functional lightweight material innovation.

## 5. Conclusion

A unit cell-based bottom-up analytical model is presented for evaluating the elastic properties of inflatable lattices (including the realistic effect of non-rigid joints), wherein a dramatic increase in the specific elastic moduli can be achieved (approx. 10^3^ times compared with conventional solid lattices). The increased values of specific Young’s and shear moduli would lead to lightweight structures with high specific stiffness, besides possessing a wide range of non-functional features. The specific elastic moduli in this novel class of inflatable lattices can further be enhanced by considering reinforced fabrics at the beam level and further involving other beam-level architectures. In principle, the generic approach concerning the hierarchical mechanics of inflatable lattices presented here can be extended to any form of two- and three-dimensional lattices considering appropriate unit cells. The current work is presented considering small deformation assumptions since the concept of inflatable lattices is introduced for the first time here. The proposed computational framework can further be extended to such large deformation regimes in the near future.

## Data Availability

All the relevant codes and data are provided along with the submission. These are also uploaded in Dryad: https://doi.org/10.5061/dryad.2ngf1vhvr [97]. Supplementary material is available online [98].
